# Supplementary material for: Dyslipidemia among adult HIV patients on antiretroviral therapy and its association with age and body mass index in Ethiopia: A systematic review and meta-analysis
Source: PLoS One. 2024 May 9;19(5):e0298525. doi: 10.1371/journal.pone.0298525 (PMC11081291; doi:10.1371/journal.pone.0298525)
Supplement: S2 Table — New Castel Ottawa was used. (DOCX) [file pone.0298525.s002.docx]

**Methodological quality assessment**

**Supplementary file 1:** Methodological quality assessment of included studies using modified Newcastle - Ottawa Scale (NOS)

| **Corresponding author** |  | **Criteria** | | | | | | | |  |
| --- | --- | --- | --- | --- | --- | --- | --- | --- | --- | --- |
|  |  | **Selection** | | | | **Comparability** | | **Outcome** | |  |
|  | **Study Design** | **Representativeness of the sample** | **Sample size** | **Non –responders** | **Ascertainment of exposure/risk factor** | **The study controls for the most important factor** | **The study control for any additional factor** | **Assessment of the outcome** | **Statistical test** | **Quality score** |
| Kemal et al | Cross-sectional | A* | **A*** | **A*** | **A*** | **-** | **A*** | A* | **A*** | **7** |
| Gebrie et al | Cross-sectional | **A*** | **A*** | **A*** | **A*** | **-** | **A*** | A* | **A*** | **7** |
| Belay E. et al | Cross-sectional | A* | **_** | **A*** | **A*** | **A*** | **A*** | A* | **A*** | **7** |
| Yazei TS | Prospective Cohort | - | **-** | **A*** | **A*** | **A*** | **A*** | A* | **A*** | **6** |
| Tadewos A. et al | Cross-sectional | A* | **A*** | **A*** | **A*** | **A*** | **A*** | A* | **A*** | **8** |
| Berhan T. et al | Cross-sectional | A* | **A*** | **A*** | **A*** | **-** | **A*** | A* | **A*** | **7** |
| Tadewos and Assegu | Prospective Cohort | - | **-** | **A*** | **A*** | **A*** | **A*** | A* | **A*** | **6** |
| Fiseha T. et al | Cross-sectional | A* | **A*** | **A*** | **A*** | **A*** | **A*** | A* | **A*** | **8** |
| Habtamu WB. et al | Cross- seccional | A* | **A*** | **A*** | **A*** | **A*** | **A*** | A* | **A*** | **8** |
| Tesfaye et al | Cross seccional | A* | **A*** | **A*** | **A*** | **A*** | **A*** | A* | **A*** | **8** |
| Abebe et al | Cross seccional | A* | **-** | **A*** | **A*** | **A*** | **-** | A* | **A*** | **6** |
| Muche Belete A. et al | cros-seccional | A* | **-** | **A*** | **A*** | **A*** | **A*** | A* | **A*** | **7** |
| Bune et al | Cross- seccional | A* | **A*** | **A*** | **A*** | **A*** | **A*** | A* | **A*** | **8** |
| Ataro, Z. and Ashenafi, W. | Cross seccional | A* | **-** | **A*** | **A*** | **A*** | **-** | A* | **A*** | **6** |

*Note: from each item account point. (Accept the study for each study design based on total score of ≥50%)*

Selection: (Maximum 5 stars)
1) Representativeness of the sample: a) Truly representative of the average in the target population. * (all subjects or random sampling) .b) Somewhat representative of the average in the target population. * (nonrandom sampling) .c) Selected group of users.d) No description of the sampling strategy.
2) Sample size:a) Justified and satisfactory. *.b) Not justified.
3) Non-respondents: a) Comparability between respondents and non-respondents characteristics is
established, and the response rate is satisfactory. * .b) The response rate is unsatisfactory, or the comparability between respondents
and non-respondents is unsatisfactory. c) No description of the response rate or the characteristics of the responders and
the non-responders.
4) Ascertainment of the exposure (risk factor): a) validated measurement tool. ** .b) Non-validated measurement tool, but the tool is available or described.* c) No description of the measurement tool.
Comparability: (Maximum 2 stars)
1) The subjects in different outcome groups are comparable, based on the study design or analysis. Confounding factors are controlled. a) The study controls for the most important factor (select one). * b) The study control for any additional factor. *
Outcome: (Maximum 3 stars)
1) Assessment of the outcome: a) Independent blind assessment. **,b) Record linkage. **,c) Self report. *,d) No description.
2) Statistical test:a) The statistical test used to analyze the data is clearly described and appropriate, and the measurement of the association is presented, including confidence intervals and the probability level (p value). *,b) The statistical test is not appropriate, not described or incomplete
