# Supplementary material for: Dyslipidemia among adult HIV patients on antiretroviral therapy and its association with age and body mass index in Ethiopia: A systematic review and meta-analysis
Source: PLoS One. 2024 May 9;19(5):e0298525. doi: 10.1371/journal.pone.0298525 (PMC11081291; doi:10.1371/journal.pone.0298525)
Supplement: S3 Table — Hoy score was used. (DOCX) [file pone.0298525.s003.docx]

**Supplementary file 2:** The risk of bias assessment tool for the included studies.

| **Corresponding author** | Representation | Sampling | Random selection | Non-response bias | Data collected | Case definition | Reliability &validity of tool | Mode of data collection | Length prevalence period | Numerator & denominator | The overall risk of bias |
| --- | --- | --- | --- | --- | --- | --- | --- | --- | --- | --- | --- |
| Kemal et al | No | Yes | **Yes** | **Yes** | **Yes** | **Yes** | **Yes** | Yes | **Yes** | **Yes** | Low risk |
| Gebrie et al | No | Yes | **Yes** | **Yes** | **Yes** | **Yes** | **Yes** | Yes | **Yes** | **Yes** | Low risk |
| Belay E. et al | **No** | **No** | **No** | **Yes** | **Yes** | **Yes** | **Yes** | Yes | **Yes** | **Yes** | Moderate risk |
| Yazie T. | No | No | **No** | **Yes** | **Yes** | **Yes** | **Yes** | Yes | **Yes** | **Yes** | Moderate risk |
| Tadewos A. et al | No | Yes | **Yes** | **Yes** | **Yes** | **Yes** | **Yes** | Yes | **Yes** | **Yes** | Low risk |
| Berhan T. et al | No | Yes | **No** | **Yes** | **Yes** | **Yes** | **Yes** | Yes | **Yes** | **Yes** | Moderate risk |
| Tadewos and Assegu | No | Yes | **No** | **Yes** | **Yes** | **Yes** | **Yes** | Yes | **Yes** | **Yes** | Low risk |
| Fiseha T. et al | Yes | Yes | **No** | **Yes** | **Yes** | **Yes** | **Yes** | Yes | **Yes** | **Yes** | Low risk |
| Habtamu WB. et al | Yes | Yes | **No** | **Yes** | **Yes** | **Yes** | **Yes** | Yes | **Yes** | **Yes** | Low risk |
| Tesfaye et al | Yes | Yes | **Yes** | **Yes** | **Yes** | **Yes** | **Yes** | Yes | **Yes** | **Yes** | Low risk |
| Abebe et al | No | No | **No** | **Yes** | **Yes** | **Yes** | **Yes** | Yes | **Yes** | **Yes** | Moderate risk |
| Muche Belete A. et al | No | Yes | **No** | **Yes** | **Yes** | **Yes** | **Yes** | Yes | **Yes** | **Yes** | Low risk |
| Bune et al | Yes | Yes | **No** | **Yes** | **Yes** | **Yes** | **Yes** | Yes | **Yes** | **Yes** | Low risk |
| Ataro, Z. and Ashenafi, W. | Yes | Yes | **No** | **Yes** | **Yes** | **Yes** | **Yes** | Yes | **Yes** | **Yes** | Low risk |
| Tilahun A. et al | No | Yes | **Yes** | **Yes** | **Yes** | **Yes** | **Yes** | Yes | **Yes** | **Yes** | Low risk |
| Assefa A. et al. | Yes | Yes | **Yes** | **Yes** | **Yes** | **Yes** | **Yes** | Yes | **Yes** | **Yes** | Low risk |
| Weldeyes E. et al. | Yes | Yes | **Yes** | **Yes** | **Yes** | **Yes** | **Yes** | Yes | **Yes** | **Yes** | Low risk |

- **Note**

Risk of bias assessment tool: Yes (low risk); No (high risk)

1. Representation: Was the study population a close representation of the national population?

2. Sampling: Was the sampling frame a true or close representation of the target population?

3. Random selection: Was some form of random selection used to select the sample OR was a census undertaken?

4. Non-response bias: Was the likelihood of non-response bias minimal?

5. Data collection: Were data collected directly from the subjects?

6. Case definition: Was an acceptable case definition used in the study?

7. Reliability and validity of study tool: Was the study instrument that measured the parameter of interest show to have reliability and validity?

8. Data collection: Was the same mode of data collection used for all subjects?

9. Prevalence period: Was the length of the prevalence period for the parameter of interest appropriate?

10. Numerators and denominators: Were the numerator(s) and denominator(s) for the parameter of interest appropriate?

The overall risk of bias scored based on the number of high risk of bias per study: low risk (≥8), moderate risk (5–7), and high risk (≤4)
