# Supplementary material for: Dyslipidemia among adult HIV patients on antiretroviral therapy and its association with age and body mass index in Ethiopia: A systematic review and meta-analysis
Source: PLoS One. 2024 May 9;19(5):e0298525. doi: 10.1371/journal.pone.0298525 (PMC11081291; doi:10.1371/journal.pone.0298525)
Supplement: S4 Table — (DOCX) [file pone.0298525.s004.docx]

HIV OR HIV OR human immunodeficiency virus OR acquired immunodeficiency syndrome AND highly active antiretroviral therapy OR HAART OR ART OR antiretroviral therapy OR protease inhibitor OR NNRTI OR NRTI OR Integrase inhibitor OR PI OR PIs OR lopinavir OR ritonavir OR lamivudine OR zidovudine OR stavudine OR nevirapine OR efavirenz OR tenofovir OR emtricitabine OR atazanavir OR darunavir AND dyslipidemias OR hypercholesterolemia OR hypertriglyceridemia OR total cholesterol OR TC OR cholesterol blood level OR triglyceride OR TG OR high-density lipoprotein cholesterol OR HDL-c OR low-density lipoprotein cholesterol OR LDL-c OR non-HDL AND Ethiopia

HIV AND HAART OR ART OR antiretroviral therapy AND dyslipidemia AND Ethiopia

HIV AND HAART OR ART OR antiretroviral therapy AND hypercholesterolemia OR total cholesterol OR cholesterol blood level AND Ethiopia

HIV AND protease inhibitor OR NNRTI OR NRTI OR Integrase inhibitor OR PI OR PIs OR lopinavir OR ritonavir OR lamivudine OR zidovudine OR stavudine OR nevirapine OR efavirenz OR tenofovir OR emtricitabine OR atazanavir OR darunavir AND dyslipidemia AND Ethiopia

| Data base searched | Terms and Keywords used | Filtering description | Results | Total results |
| --- | --- | --- | --- | --- |
| PubMed | (((HIV OR HIV OR human immunodeficiency virus OR acquired immunodeficiency syndrome) AND (highly active antiretroviral therapy OR HAART OR ART OR antiretroviral therapy OR protease inhibitor OR NNRTI OR NRTI OR Integrase inhibitor OR PI OR PIs OR lopinavir OR ritonavir OR lamivudine OR zidovudine OR stavudine OR nevirapine OR efavirenz OR tenofovir OR emtricitabine OR atazanavir OR darunavir)) AND (dyslipidemias OR hypercholesterolemia OR hypertriglyceridemia OR total cholesterol OR TC OR cholesterol blood level OR triglyceride OR TG OR high-density lipoprotein cholesterol OR HDL-c OR low-density lipoprotein cholesterol OR LDL-c OR non-HDL)) AND (Ethiopia) |  | 41 |  |
|  | Ethiopia |  | 39041 |  |
|  | dyslipidemias OR hypercholesterolemia OR hypertriglyceridemia OR total cholesterol OR TC OR cholesterol blood level OR triglyceride OR TG OR high-density lipoprotein cholesterol OR HDL-c OR low-density lipoprotein cholesterol OR LDL-c OR non-HDL |  | 513, 854 |  |
|  | highly active antiretroviral therapy OR HAART OR ART OR antiretroviral therapy OR protease inhibitor OR NNRTI OR NRTI OR Integrase inhibitor OR PI OR PIs OR lopinavir OR ritonavir OR lamivudine OR zidovudine OR stavudine OR nevirapine OR efavirenz OR tenofovir OR emtricitabine OR atazanavir OR darunavir |  | 753, 584 |  |
|  | HIV OR HIV OR human immunodeficiency virus OR acquired immunodeficiency syndrome |  | 467, 139 |  |
|  |  |  |  |  |
| Google scholar  (Advanced search) |  | ALL | 56 | 56 |
| Science direct  (Advance search) | HIV AND antiretroviral therapy AND dyslipidemia AND Ethiopia | Limited to research articles article type, 2 publication title and 2 subject areas | 14 | 14 |
| DOAG | HIV AND antiretroviral therapy AND dyslipidemia AND Ethiopia | All fields | 8 | 8 |
| WorldCat | HIV OR HIV OR human immunodeficiency virus OR acquired immunodeficiency syndrome AND highly active antiretroviral therapy OR HAART OR ART OR antiretroviral therapy OR protease inhibitor OR NNRTI OR NRTI OR Integrase inhibitor OR PI OR PIs OR lopinavir OR ritonavir OR lamivudine OR zidovudine OR stavudine OR nevirapine OR efavirenz OR tenofovir OR emtricitabine OR atazanavir OR darunavir AND dyslipidemias OR hypercholesterolemia OR hypertriglyceridemia OR total cholesterol OR TC OR cholesterol blood level OR triglyceride OR TG OR high-density lipoprotein cholesterol OR HDL-c OR low-density lipoprotein cholesterol OR LDL-c OR non-HDL AND Ethiopia | Downloadable articles published in English |  |  |
| WorldCat |  |  |  |  |
|  | HIV AND HAART OR ART OR antiretroviral therapy AND dyslipidemia AND Ethiopia | Articles | 34 |  |
|  | HIV AND HAART OR ART OR antiretroviral therapy AND hypercholesterolemia OR total cholesterol OR cholesterol blood level AND Ethiopia | Downloadable published article in English | 44 |  |
|  | HIV AND protease inhibitor OR NNRTI OR NRTI OR Integrase inhibitor OR PI OR PIs OR lopinavir OR ritonavir OR lamivudine OR zidovudine OR stavudine OR nevirapine OR efavirenz OR tenofovir OR emtricitabine OR atazanavir OR darunavir AND dyslipidemia AND Ethiopia |  | 31 |  |
|  |  |  |  |  |
|  |  |  |  |  |
