# Supplementary material for: Dyslipidemia among adult HIV patients on antiretroviral therapy and its association with age and body mass index in Ethiopia: A systematic review and meta-analysis
Source: PLoS One. 2024 May 9;19(5):e0298525. doi: 10.1371/journal.pone.0298525 (PMC11081291; doi:10.1371/journal.pone.0298525)
Supplement: Attachment — Submitted filename: Author response to reviewers 2.docx [file pone.0298525.s005.docx]

PLOS ONE Decision: Revision required [PONE-D-23-28119] - [EMID:eae76a4b619b03df]

PONE-D-23-28119

Dyslipidemia among adult HIV patients on antiretroviral therapy and its association with age and body mass index in Ethiopia: A Systematic Review and meta-analysis.

PLOS ONE

**Author response to Reviewer and Academic Editor’s comments**

Dear Academic Editor and reviewer, we would like to thank you for all your effort and invaluable comments to improve the manuscript. We have done all the best to address both the Academic Editor and reviewer’s comments. All revisions were made as per the comments given. All changes have been highlighted in the manuscript labelled as “Revised Manuscript with Track Changes”.

We thank you all again!

Journal requirements:

When submitting your revision, we need you to address these additional requirements.

1. Please ensure that your manuscript meets PLOS ONE's style requirements, including those for file naming. The PLOS ONE style templates can be found at

<https://journals.plos.org/plosone/s/file?id=wjVg/PLOSOne_formatting_sample_main_body.pdf> and

<https://journals.plos.org/plosone/s/file?id=ba62/PLOSOne_formatting_sample_title_authors_affiliations.pdf>

Author response: Dear Editor, we have read the journal requirements via the links given above and we have made amendments that have been highlighted in the “revised manuscript with track changes”.

2. When completing the data availability statement of the submission form, you indicated that you will make your data available on acceptance. We strongly recommend all authors decide on a data sharing plan before acceptance, as the process can be lengthy and hold up publication timelines. Please note that, though access restrictions are acceptable now, your entire data will need to be made freely accessible if your manuscript is accepted for publication. This policy applies to all data except where public deposition would breach compliance with the protocol approved by your research ethics board. If you are unable to adhere to our open data policy, please kindly revise your statement to explain your reasoning and we will seek the editor's input on an exemption. Please be assured that, once you have provided your new statement, the assessment of your exemption will not hold up the peer review process.

Author response: Dear Editor, we thank again for the comment. All data used for this systematic review and meta-analysis are available within the manuscript and /or supplementary file. Therefore, the data availability statement in the online form and in the manuscript is “All data generated and analysed during this study are included in this published article (and its supplementary file)”.

[Note: HTML markup is below. Please do not edit.
Reviewers' comments:
Reviewer's Responses to Questions

**Comments to the Author**

1. Is the manuscript technically sound, and do the data support the conclusions?

The manuscript must describe a technically sound piece of scientific research with data that supports the conclusions. Experiments must have been conducted rigorously, with appropriate controls, replication, and sample sizes. The conclusions must be drawn appropriately based on the data presented.

Reviewer #1: Partly

Author response #1: Dear reviewer, we made revision to the conclusion and the change has been highlighted in the “revised manuscript with track changes”

2. Has the statistical analysis been performed appropriately and rigorously?

Reviewer #1: Yes

Author response#2: Thank you

3. Have the authors made all data underlying the findings in their manuscript fully available?

The [PLOS Data policy](http://www.plosone.org/static/policies.action#sharing) requires authors to make all data underlying the findings described in their manuscript fully available without restriction, with rare exception (please refer to the Data Availability Statement in the manuscript PDF file). The data should be provided as part of the manuscript or its supporting information, or deposited to a public repository. For example, in addition to summary statistics, the data points behind means, medians and variance measures should be available. If there are restrictions on publicly sharing data—e.g. participant privacy or use of data from a third party—those must be specified.

Reviewer #1: Yes

Author response#3: Thank you again for your suggestion.

4. Is the manuscript presented in an intelligible fashion and written in standard English?

PLOS ONE does not copyedit accepted manuscripts, so the language in submitted articles must be clear, correct, and unambiguous. Any typographical or grammatical errors should be corrected at revision, so please note any specific errors here.

Reviewer #1: Yes

Author response#4: Thank you again

5. Review Comments to the Author

Please use the space provided to explain your answers to the questions above. You may also include additional comments for the author, including concerns about dual publication, research ethics, or publication ethics. (Please upload your review as an attachment if it exceeds 20,000 characters)

Reviewer #1: I have read this manuscript with huge interest. Dyslipidaemia contributes to the huge cardiovascular burden in HIV seropositive patients. The authors attempted to carry out a review and meta-analysis of local Ethiopian data on dyslipidaemia in HIV positive patients.
HIV infection is a multi-system disease and the presence of opportunistic infections, immunological status (typified by CD4 count levels), viral load, drugs (both HAART and medications used to treat other illnesses) add to the burden of the disease. The authors described local dyslipidaemia prevalence rates and how HAART affect lipid fractions. They also found that dyslipidaemia had significant relationships with age and BMI.
The authors are advised to relate dyslipidaemia with co-existing opportunistic illnesses, other drugs (apart from HAART which affect lipids), CD4 count and viral load from the available data as this will add more depth to the write up.

Author response#5: Dear reviewer, your insightful comments have been instrumental in refining our work.

The relationship between viral load and dyslipidemia among people living with HIV/AIDS (PLWHA) who are on highly active antiretroviral therapy (HAART) is a complex and multifaceted one. High viral load is associated with chronic inflammation in the body, which can contribute to dyslipidemia by decreasing lipoprotein lipase activity and increasing cholesterol synthesis. HIV replication activates the immune system, leading to the production of inflammatory cytokines like C-reactive protein (CRP) and interleukin-6 (IL-6). These markers can further interfere with lipid metabolism and promote dyslipidemia.

In response to your suggestions, we have made revisions on CD4, non-ART medication, substance use and comorbidities in the result and discussion sections to provide a more comprehensive description of dyslipidemia. We found that dyslipidemia was associated with low CD4 count, substance use and comorbidity like cancer. While no studies reported on the association between dyslipidemia with opportunistic infection and viral load. We have explicitly addressed this limitation in our manuscript, highlighting the absence of relevant studies on the association between dyslipidemia with opportunistic infections and viral load. Kindly see the revised manuscript.

There are also a lot of grammatical and syntax errors in the manuscript which the authors should address.

Author response#6: Dear reviewer, we had read the whole manuscript and made necessary changes that highlighted in the “revised manuscript with track changes”.

These changes should be made and the manuscript resubmitted.

Author response#6: Dear reviewer, necessary revisions have been made as per your comments and changes have been highlighted in the “revised manuscript with track changes”
